# K-Wire-Based External Fixator for Management of Salter-Harris Type-II Distal Femur Physeal Injury

**DOI:** 10.7759/cureus.46070

**Published:** 2023-09-27

**Authors:** Siddhartha Sinha, Neel Aggarwal, Arvind Kumar, Lokendra Singh, Asif Iqbal, Owais A Qureshi, Sandeep Kumar, Javed Jameel

**Affiliations:** 1 Orthopaedics, Hamdard Institute of Medical Sciences and Research, New Delhi, IND; 2 Orthopaedics, Jai Prakash Narayan Apex Trauma Centre (JPNATC) All India Institute of Medical Sciences, New Delhi, IND

**Keywords:** salter-harris, physeal slip, physeal injuries, k wire-based external fixation, joshi external stabilization system, external fixation, disal femur physeal slip

## Abstract

We present a case of a 16-year-old male with a Salter-Harris type II physeal slip of the distal femur managed with closed reduction and K wire and clamp-based external fixator. Knee range of motion exercises were initiated after one week. The union was observed at 10 weeks, and implant removal was done on an outpatient basis. At one year follow-up, the patient had good clinical and radiological outcomes. The K-wire-based external fixator frame is an effective fixation method for distal femur physeal slips in older children, providing favorable radiological and functional outcomes.

## Introduction

Distal femur physeal injuries are relatively uncommon, comprising about 1% of total physeal injuries [1]. Due to its rare nature, the management of such injuries is not well described and often leads to controversy. The distal femur physis is responsible for 70% of growth for the femoral length, and the chances of growth arrest, residual deformity, and other complications are high, further complicating the management [2]. There are limited fixation options for the fixation of these injuries. To the best of our knowledge, there is no description of such injuries being managed using K-wire-based external fixator devices. We present a case of a 16-year-old male child with a Salter-Harris type 2 physeal injury managed with a K-wire-based external stabilization system. 

## Case presentation

**Figure 1:**
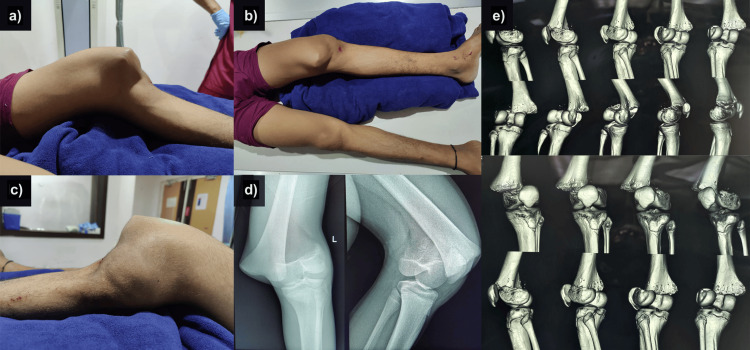
Clinical findings at the time of presentation (a-c); X-ray shows a Salter-Harris type II physeal slip (d); computed tomography scan showing the distal femoral physeal slip (e).

A 16-year-old male child presented to the casualty with complaints of pain, swelling deformity around the left knee, and inability to move the left lower limb following an alleged fall from a two-wheel motorcycle. There was no history of loss of consciousness, seizures, abdominal pain, weakness, or loss of sensations in the upper or lower limbs. On examination, there was a prominent swelling along the medial border of the thigh and leg sagging posteriorly. An associated displacement of the patella on the lateral side along with the tibia was also seen (Figures 1A-1C). On palpation, the medial swelling was tender, bony hard, and continuous with the femur. The normal anatomical relationship between the femur, patella and tibia was lost. Range of motion was not attempted, the distal pulses were palpable, and the patient could move his toes actively. The limb was splinted, and radiological examination revealed a Salter-Harris type II physeal slip (Figures 1D-1E).

**Figure 2:**
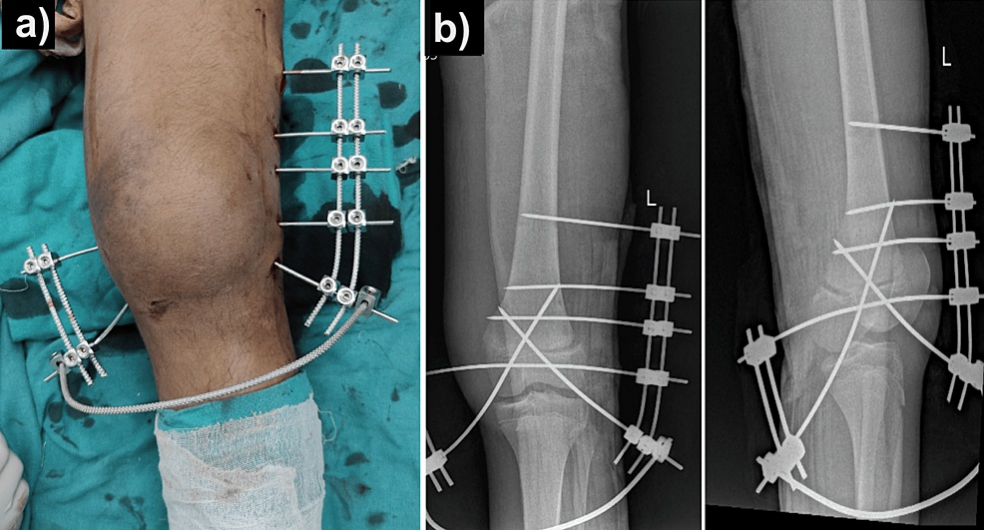
Closed reduction internal fixation of the distal femoral physeal slip with K-wire and clamp-based external fixator construct: clinical photograph (a), post-operative radiological images (b).

The patient was then shifted to the operating theatre, where the closed reduction was done using two Steinmann pins as joysticks under spinal anesthesia. Once acceptable reduction was achieved, the physeal slip was stable and provisional reduction was done using 2.5 mm K-wires in a cross configuration. A K-wire-based external fixator system (Joshi external stabilization system [JESS]) was then constructed over the K-wires after pre-tensioning the wires to produce a stable construct (Figures 2A, 2B). 

**Figure 3:**
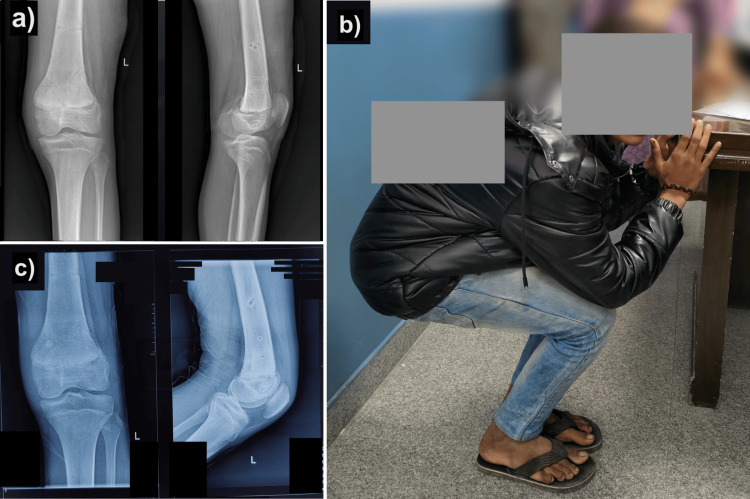
X-ray showing union in an acceptable position at 10 weeks after external-fixator removal (a); the patient could flex knees to 90° at 10 weeks post-operative (b); X-ray showing union at one-year post-operative period (c).

Knee range of motion and quadriceps strengthening exercises were started on the second postoperative day, and the patient was mobilized on non-weight bearing walker mobilization. Knee range of motion at two weeks was 0-30 degrees, and by eight weeks, it was 0-90 degrees on the fixator. Repeat X-ray showed union in an acceptable position, and the patient was planned for implant removal. At 10 weeks post-op, the patient walked without support and had a knee flexion of 90 degrees (Figures 3A-3B). The patient was on regular follow-up, and at 12 months post-op, the patient could sit cross-legged and squat without any limb length discrepancy. X-ray showed the union in an acceptable position (Figure 3C). The Knee Society Score (KSS) was 100 (excellent) at the final follow-up. 

## Discussion

Distal femur physeal slips often result in angular deformities and limb length discrepancies [3,4]. These injuries are more common in males, often following sporting activities, falls, and road traffic accidents, and are commonly Salter-Harris type 2 or 3 injuries [3,5-7]. Conservative management options are not preferred due to increased rates of loss of reduction and, eventually, manipulation [5]. Many treatment options have been described for the management of distal femur physeal slips ranging from smooth K-wires, Steinmann pin, screw fixation, and pediatric slide traction plates, have been described [3-8]. Most are type 2 injuries with high complication rates up to 36% [3]. Complications include growth arrest, limb length discrepancy and angular deformities, physeal bridge, injury to ligaments and menisci, peroneal neuropraxia and popliteal vessel injury [1,3-5,9]. 

Closed reduction is preferred for management, but up to 46% require open reduction [4]. Most authors have reported using percutaneous pinning, screw fixation, and crossed smooth K-wires after reduction for fixation. Non-physeal sparing techniques (fixation using Steinmann pins) have been reported to have higher complication rates but have not been found to be clinically significant [4,10]. The role of external fixation and its outcomes are not well explored in the literature. The method of external fixation has been described in a dog and rabbit using a circular fixator [11,12].

JESS is a versatile external fixation system based on K-wires, link clamps and rods. It works on the principle of distraction histiogenesis and provides dynamic, stable fixation. It was initially designed for hand injuries, but with time, its range of indications has been expanding in trauma, deformity correction, and plastic surgery. The frame allows for movement in the postoperative period, allowing better functional outcomes. The advantages of using this system for distal femoral physeal slips were that we were able to perform the reduction in a closed manner and hold the reduction in an acceptable position using the JESS construct, mobilize the patient early and avoid a long leg plaster, which would have otherwise led to knee stiffness. The K-wires spanning the physis were smooth and did not damage the physis. The frame was constructed in a manner that allowed for the initiation of the range of motion at the knee joint without compromising the stability of the fixation. The primary disadvantages of this construct are the risk of pin site infection, loosening of the construct and additional procedures for frame removal. Although we didn't encounter the complication of physeal arrest at one-year follow-up, the risk of physeal arrest is definite, and care should be taken to avoid excessive manipulation at the physeal region. The lack of long-term follow-up is one major limitation of this case study.

## Conclusions

K-wire-based external fixator constructs are an acceptable mode of treatment for Salter-Harris type II physeal injuries of the distal femur as they provide good clinical, functional and radiological outcomes. The fixator is a less invasive mode of fixation and can be preferred for physeal injuries in which plate or nail osteosynthesis can create growth problems owing to open physis. The method can also be replicated for other physeal injuries of the lower limbs and doesn't need any major surgical procedure for implant removal.
